# *Plasmodium vivax* transcriptional profiling of low input cryopreserved isolates through the intraerythrocytic development cycle

**DOI:** 10.1371/journal.pntd.0008104

**Published:** 2020-03-02

**Authors:** Gabriel W. Rangel, Martha A. Clark, Usheer Kanjee, Jonathan M. Goldberg, Bronwyn MacInnis, Maria José Menezes, Marcelo U. Ferreira, Manoj T. Duraisingh

**Affiliations:** 1 Department of Immunology and Infectious Diseases, Harvard T.H. Chan School of Public Health, Boston, Massachusetts, United States of America; 2 Broad Institute of Harvard and MIT, Cambridge, Massachusetts, United States of America; 3 Department of Parasitology, Institute of Biomedical Sciences, University of São Paulo, São Paulo, Brazil; Deakin University, AUSTRALIA

## Abstract

Approximately one-third of the global population is at risk of *Plasmodium vivax* infection, and an estimated 7.51 million cases were reported in 2017. Although, *P*. *vivax* research is currently limited by the lack of a robust continuous *in vitro* culture system for this parasite, recent work optimizing short-term *ex vivo* culture of *P*. *vivax* from cryopreserved isolates has facilitated quantitative assays on synchronous parasites. Pairing this improved culture system with low-input Smart-seq2 RNAseq library preparation, we sought to determine whether transcriptional profiling of *P*. *vivax* would provide insight into the differential survival of parasites in different culture media. To this end we probed the transcriptional signature of three different *ex vivo P*. *vivax* samples in four different culture media using only 1000 cells for each time point taken during the course of the intraerythrocytic development cycle (IDC). Using this strategy, we achieved similar quality transcriptional data to previously reported *P*. *vivax* transcriptomes. We found little effect with varying culture media on parasite transcriptional signatures, identified many novel gametocyte-specific genes from transcriptomes of FACS-isolated gametocytes, and determined invasion ligand expression in schizonts in biological isolates and across the IDC. In total, these data demonstrate the feasibility and utility of *P*. *vivax* RNAseq-based transcriptomic studies using minimal biomass input to maximize experimental capacity.

## Author summary

*Plasmodium vivax* is the most prevalent malaria-causing parasite species outside of Sub-Saharan Africa and has many unique and poorly understood biological characteristics that make it particularly challenging to study and combat. Transcriptomic profiling of *P*. *vivax* under various conditions has the potential to unlock new experimental abilities and aid in elucidating biology and the development of clinical interventions. However, a lack of a robust *in vitro* culture system for this parasite has restricted transcriptomic studies to researchers with timely access to fresh human isolates from clinics, which often are in resource-poor settings, as well as nearby, well-equipped laboratories for sample processing. This study aimed to gain insight into the differential survival of *P*. *vivax* in various culture media from the parasites transcriptional signature in each media. By implementing low-input RNA library preparation strategies, this study obtains robust transcriptomic data at various parasite development stages and in different culture conditions from just 1000 FACS-purified, *P*. *vivax*-infected erythrocytes from viable cryopreserved patient isolates. With these data, we find culture media has little effect on transcriptional profile, we characterize invasion ligand expression across intraerythrocytic development and between clinical isolates, and we define the transcriptome of sexual, transmissible stages of the *P*. *vivax* parasite. These results highlight the establishment and utility of a powerful platform for studying the transcriptomic biology of this particularly challenging parasite.

## Introduction

Although malaria-causing parasites, including *P*. *falciparum* and *P*. *vivax*, were discovered nearly 130 years ago, and the blood stages of *P*. *falciparum*, the deadliest species of malaria parasite, has been culture-adapted since 1976, continuous *P*. *vivax* blood-stage culture remains elusive, significantly hindering experimentation and the development of new therapeutic strategies [1,2]. This contributes to the fact that *Plasmodium vivax* remains a significant global health problem, putting approximately one-third of the global population at risk for infection and resulting in an estimated 7.51 million reported cases in 2017 [3]. Furthermore, relative to other malaria species, *P*. *vivax* has many unique and challenging biological qualities including the presence of spontaneously reactivating dormant liver stages (hypnozoites), strong preference for invading the youngest circulating host red blood cells (reticulocytes), and the rapid development of transmissible gametocytes often before clinical symptoms present [4].

The absence of an in vitro culture system for studying the aforementioned unique aspects of *P*. *vivax* biology represent a major hindrance to the development of effective interventions against *P*. *vivax*. Our group has recently demonstrated that various culture media formulations have significant impact on the survival of *P*. *vivax*, and that the use of Iscove’s Modified Dulbecco’s Medium (IMDM) boosts *ex vivo P*. *vivax* survival for short-term culture, expanding the utility of small-volume, cryopreserved isolates [5]. The underlying mechanisms governing the improved survival of the parasite remain unclear.

Characterizing the transcriptomic profiles of various stages of this parasite has potential to yield important advancements in understanding the biology underlying these unique traits, thereby enabling many new experimental studies and public health interventions. Indeed, transcriptome profiles of *P*. *vivax* across the intraerythrocytic development cycle (IDC) have been generated using both microarray and RNAseq approaches, but because of the lack of a continuous *in vitro* culture system for the IDC of this parasite, these studies have been either limited to characterizing IDC stages immediately available from the patient [6–8], or have been limited to research groups that have access to both fresh, clinical isolates as well as proximal, well-equipped laboratory space for sample processing [9,10]. Furthermore, while some of these publications report the robust transcriptomic characterization of patient isolates of *P*. *vivax* through the 48-hour IDC, to date, these studies have required large amounts of fresh starting material (10–20 mL whole blood samples), and these study designs are not able to isolate transcriptomes of sexual-stage from asexual-stage intraerythrocytic parasites, a distinction that would greatly enable understanding the unique *P*. *vivax* gametocyte biology and the development of transmission-blocking interventions [9].

One reason a relatively large amount of starting material is required for these studies is the high rate of loss of *P*. *vivax* parasitemia through the course of the IDC when using McCoy’s 5A culture media [5,11]. In recent work, we demonstrated that various culture media formulations have significant impact on the survival of *P*. *vivax*, and that the use of Iscove’s Modified Dulbecco’s Medium (IMDM) boosts *ex vivo P*. *vivax* survival for short-term culture, expanding the utility of small-volume, cryopreserved isolates [5].

Another hurdle in generating robust transcriptional data from patient samples is the low fraction of parasite RNA in a samples relative to human RNA, with parasite transcripts often accounting for far less than half of sequenced RNA [6,8,10]. This is likely a result of the presence of host leukocytes that are collected along with the parasitized erythrocytes during patient sample acquisition. Fortunately, techniques for both filtering away leukocytes from patient samples as well as purifying *Plasmodium* parasites via fluorescence-activated cell sorting (FACS) have been established [12,13], and employing these may reduce the impact of this hurdle on acquiring strong transcriptomic data.

An additional challenge in studying the transcriptomes of various stages of the *P*. *vivax* IDC is the asynchrony of parasites in a patient sample. Indeed, it has been reported that the vast majority of transcripts characterized from parasites directly from blood-draw are from trophozoites [6,8]. This is likely because later stage parasites tend to have more transcript abundance, and because the presence of later stages of *P*. *vivax* parasites is much less likely in circulating blood compared to earlier stages [14]. However, as we reported previously, only the early IDC stages tend to survive our reported cryopreservation and thawing process, thereby facilitating the study of relatively synchronous parasite populations [5].

Lastly, recent advancements in RNA library preparation for RNAseq transcriptomic approaches have greatly improved detection, coverage, bias and accuracy for relatively small amounts of input material [15]. These advancements have the potential to enable researchers to use minimal numbers of parasites to probe *P*. *vivax* transcriptional response to environmental perturbations.

This present study capitalizes on the advancements in efficient parasite isolation, in culture of viable cryopreserved *P*. *vivax*, and in low RNA input SMARTseq2 RNAseq library preparation to determine (i) the capacity of SMARTseq2 to yield reliable *P*. *vivax* transcriptomes from just 1000 infected cells and (ii) whether by transcriptional profiling *P*. *vivax* in different media of the course of the IDC we might gain insight into the differential survival of P. vivax in different media. To this end, we generate robust transcriptomes from three cryopreserved clinical *P*. *vivax* isolates in four different culture media from just 1000, *P*. *vivax-*infected erythrocytes purified by Fluorescence-Activated Cell Sorting (FACS) at various time points throughout the IDC [5,15]. From these transcriptomes, we find minimal effect of culture media on the transcriptional profile of the parasite, we characterize invasion ligand expression across intraerythrocytic development and between clinical isolates, and we define the transcriptome of FACS-enriched sexual, transmissible stages of the *P*. *vivax* parasite.

## Methods

### Ethical approval and sample collection

Written informed consent was obtained from all study participants or their parents or guardians; assent was obtained from children aged less than 18 years. The Institutional Review Boards of the Institute of Biomedical Sciences at the University of São Paulo, Brazil (1169/CEPSH, 2014) and Harvard T. H. Chan School of Public Health (21410–101) approved the protocols for parasite sample collection. Samples were collected in Mâncio Lima, Acre State, Brazil, from *P*. *vivax* patients diagnosed via conventional thick-smear microscopy performed in the context of a randomized, open-label clinical trial [16], and the samples were depleted of leukocytes and cryopreserved as previously described [17].

### *P*. *vivax* maturation and RNA sample collection

**Fig 1:**
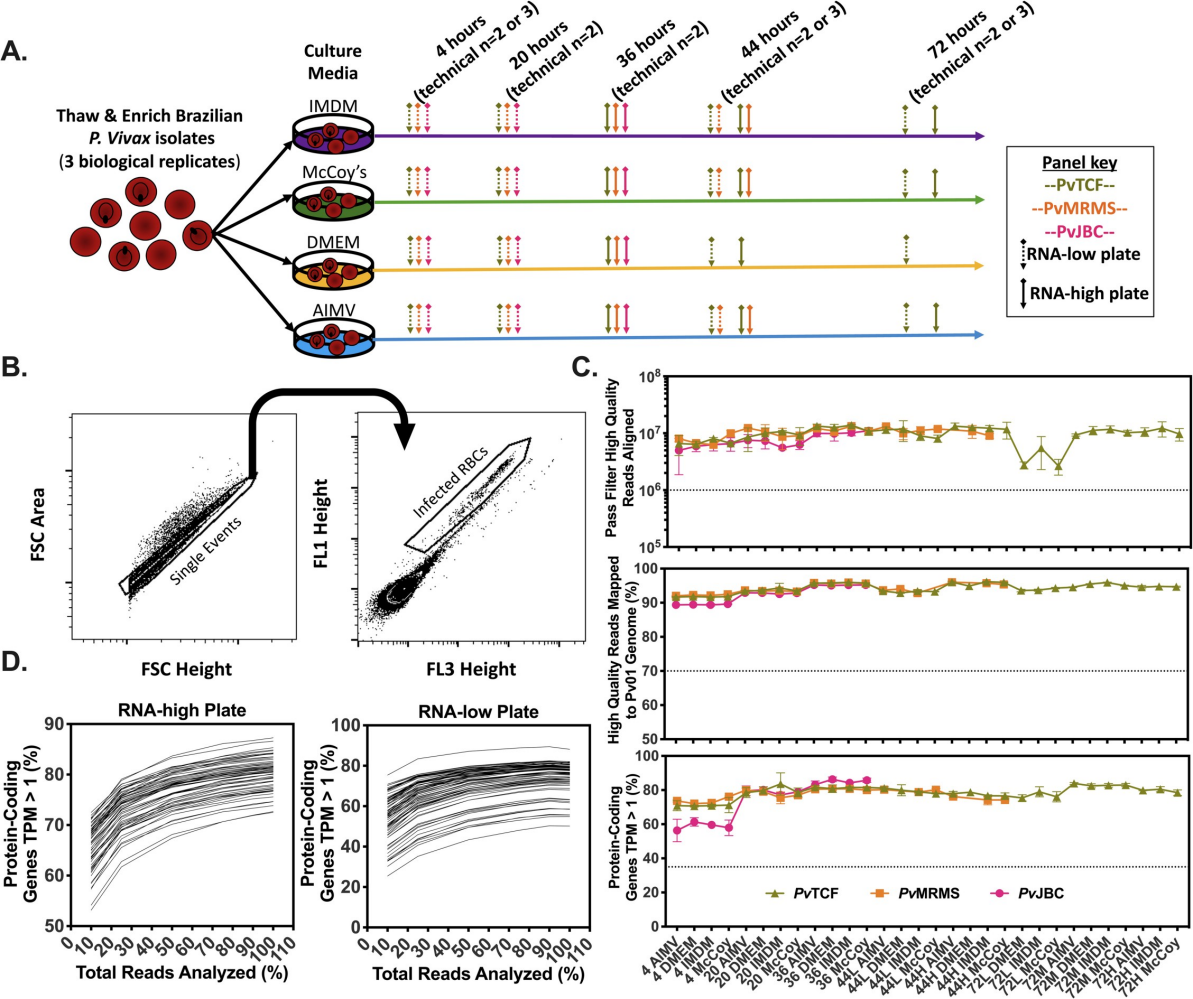
Small volume, cryopreserved *Plasmodium vivax* isolates provide robust RNAseq data via the Smart-seq2 platform. A) Experimental strategy. Three separate Brazilian *P*. *vivax* patient isolates were thawed, enriched, and cultured in four different culture media. Samples were taken at 4, 20, 36, 44 and 72 hours post-thaw for FACS, provided enough biomass remained, and were sorted based on DNA content or DNA high versus DNA-low when applicable. B) The FACS gating scheme used to separate infected RBCs from uninfected RBCs using Vybrant DyeCycle Green stain. C) Quality control analysis of each individual sample. The hashed horizontal lines represent previously established thresholds below which any particular sample would have been excluded. Error bars represent the standard deviation of technical replicates. D) Transcriptome saturation curves for each sequencing plate showing % protein-coding genes covered (TPM > 1) when 10%, 25%, 50%, 75%, 90%, and 100% of generated reads were analyzed.

We prescreened approximately 15 cryopreserved, leukofiltered blood isolates from separate Brazilian donors for acceptable recoverable parasitemia (>0.2% post-enrichment). Vials from three of the acceptable donations were thawed and enriched on a 1.080 g/mL KCl Percoll gradient as previously described [5]. The enriched parasites were plated at 10E7 cells per mL (~1% hematocrit) in indicated culture media supplemented with 10% v/v AB^+^ heat-inactivated human serum pooled from 15 human donors and 50 μg/mL gentamicin. Samples were collected at 4, 20, 36, 44 and 72 hours post-thaw in technical duplicate or triplicate, depending on available biomass, for FACS and microscopy (Fig 1A). For FACS, each technical replicate was stained with Vybrant DyeCycle Green (1:5000) for 20 minutes at 37°C. Then 1000 individual parasitized cells were sorted into lysis solution made of 1x Buffer TCL (Qiagen) with 1% v/v 2-mercaptoethanol using a BioRad S3e sorter or Sony SH800 sorter. *P*. *vivax* infected cells were selected by gating for single events then for Vybrant DyeCycle Green-positive (DNA-positive) cells (Fig 1B). Upon collection, transcriptomic samples were immediately placed on dry ice and then transferred to -80°C storage. When remaining biomass permitted, 1E5-3E5 additional cells were sorted, mixed with 2E6 uninfected “carrier” cells, and cytospins were made for staging by light microscopy. Once samples from all time points were collected, transcriptomic samples were thawed and plated onto either an RNA-low or RNA-high plate based on expected sample RNA content as indicated (Fig 1A) and submitted to the Broad Technology Labs for library preparation via the Smart-seq2 strategy using 18 cycles of PCR amplification [15]. Sequencing, which was performed on the Illumina NextSeq500 platform using High Output v2 kit. Reproducibility across flow cells was verified, and the data was concatenated before additional processing.

### Read alignment, quantification and analysis

Picard Tools v2.18.7 was used to assess library size and percent high quality mapped reads. Paired-end reads were aligned to the *Plasmodium vivax* P01 genome [18] using Spliced Transcripts Alignment to a Reference (STAR) v2.5.0c [19] with the parameters—runThreadN 16,—runMode alignReads,—twopassMode Basic,—alignIntronMax 500,—alignMatesGapMax 500,—sjdbScore 2,—quantMode TranscriptomeSAM GeneCounts,—outReadsUnmapped Fastx, and—sjdbOverhang 24. Reads were quantified and transcripts per million (TPM) were calculated using the RNAseq by Expectation-Maximization (RSEM) package v1.2.29 [20] with the command and parameters rsem-calculate-expression -p 16, paired-end,—bam, and—output-genome-bam.

Principal components (PC) analysis was performed through the *prcomp* function in the *stats* v3.4.4 package of RStudio v 1.1.447 on log_2_(TPM+1) data. The staging was categorized based on light microscopy slides of the *Pv*TCF samples taken after FACS sorting at each time point, as well as our published understanding of the progression of general *P*. *vivax* staging in relation to post-thaw timing [5], and S4 Fig has representative images of FACS-sorted parasites from isolate *Pv*TCF.

Differential gene expression analysis was performed using the Empirical Analysis of Digital Gene Expression Data in R (edgeR) package v3.20.9 [21]. Genes with at least 16-fold increase in expression, as calculated by edgeR, in the *Pv*TCF 72 hour DNA-mid subpopulation compared to the *Pv*TCF 72 hour DNA-high subpopulation were sorted by average TPM in the 72 hour DNA-mid subpopulation, as calculated by RSEM. Enriched gene ontology term tree maps of biological processes corresponding to schizont and gametocyte populations were created using REVIGO analysis of all genes significantly (Benjamini-Hochberg adjusted p-value < 0.05) upregulated in each population by at least log_2_(fold change) > 2 [22].

### Quantitative reverse transcriptase-polymerase chain reaction

qRT-PCR was performed essentially as previously described [23]. Briefly, isolate *Pv*TCF was thawed, enriched and matured as above to 72 hours post-thaw. 1E5-2E5 parasites from the DNA-high, DNA-mid and DNA-low subpopulations as well as cells from the uninfected subpopulation were FACS sorted as above into TRIzol Reagent (Thermo Fisher Scientific), frozen in liquid nitrogen and stored at -80°C. RNA isolation. cDNA synthesis was performed according to the manufacturer’s instructions using the RNeasy Micro Kit (Qiagen) and the SuperScript VILO (Thermo Fisher Scientific) respectively.

Primers to our target genes were designed using the online PrimerQuest Tool from Integrated DNA Technologies (S1 Table). PVP01_0620000 (methionine tRNA ligase) was selected as a housekeeping control gene because it has previously been successfully used for qPCR analysis of *P*. *vivax* genes [24].

qPCR was performed in technical triplicate on a StepOnePlus Real-Time PCR System (Applied Biosystems) using Fast SYBR Green PCR Master Mix (Applied Biosystems) and indicated primers according to manufacturer specifications, with these thermocycler conditions: 95°C for 20 seconds, followed by 40 cycles of 95°C for 3 seconds and 60°C for 30 seconds. Relative gene expression was calculated by the ΔΔCt method, normalizing first by the housekeeping control, then by the DNA-high sample.

### Accession numbers

P25 (PVP01_0616100), LAP5 (PVP01_1255400), MRScyt (PVP01_0620000), G377 (PVP01_1467200), PSOP12 (PVP01_1020200).

## Results

### High quality *P*. *vivax* transcriptomes achieved from low RNA input SMARTseq2 library preparation

Previously we demonstrated significant effects of culture media on *P*. *vivax ex vivo* survival [5], and because the biological explanations for these effects remain elusive, we sought to identify a transcriptional signature that may help elucidate the mechanism of differential growth in various media formulations. To this end, three cryopreserved clinical P. vivax isolates were inoculated into IMDM, DMEM, McCoy’s and AIMV media and 1,000 infected cells were collected by FACS at 4, 20, 36, 44, and 72 hours post thaw for transcriptional analysis using the low RNA input SMARTseq2 RNAseq platform.

As 1,000 ring stage malaria infected cells are estimated to contain only a fraction of the amount of RNA as a single mammalian cell [25], we first assessed the quality of these data. Various metrics including library size > 1 million pass-filter (PF) high-quality (HQ) reads, > 70 percent HQ reads mapped to the target genome (*Plasmodium vivax* P01) [18], and > 35 percent of protein coding genome covered above an empirically chosen threshold of gene expression, transcripts per million (TPM) ≥ 1 were assessed for every sample, and each passed thresholds previously established for Smart-seq2 RNAseq libraries generated from single mammalian cells [26] (Figs 1C and S1). Notably, each of our sample libraries consisted of reads of which 89%-96% (median 94%) mapped to the *Pv*P01 genome (Fig 1C). Furthermore, to assess how comprehensively the data represent the transcriptome of each sample, transcriptome saturation curves were created, and the percentage of protein-coding genes with TPM ≥ 1 for every sample largely plateaus as total reads analyzed approaches 100% (Fig 1D).

### Culture media effects on *P*. *vivax* transcriptomes

**Fig 2:**
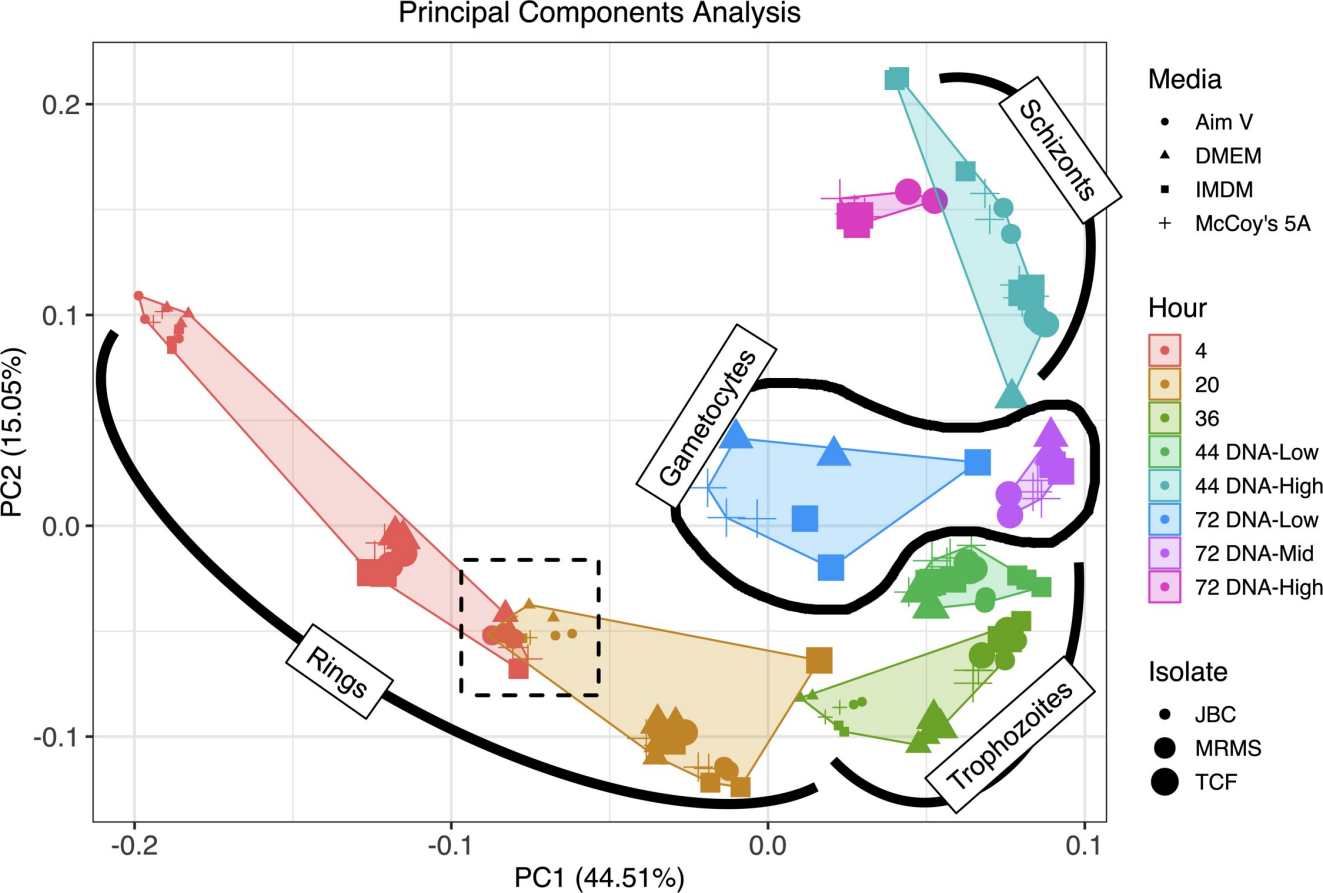
The majority of transcriptional variation is explained by IDC stage and patient isolate. A scatter plot of the first two principle components with point colors and associated polygons representing different time points of samples analyzed, point shapes representing different media used, and point size representing the different patient isolate sampled. The hashed box highlights overlap between isolate *Pv*MRMS at hour 4 post-thaw and *Pv*JBC at hour 20 post thaw.

Next we examined whether the transcriptional signature of *P*. *vivax* varied between parasites grown in different culture media. To reduce the data complexity and visually compare the relatedness of each sample we performed principal components (PC) analysis on our transcriptomes and found that approximately 60% of variation in transcriptional signatures across samples can be accounted for by the first two principal components. PC1 (44.51% of sample variation) is driven by sample time point through the IDC and PC2 (15.05% of sample variation) is explained by transcriptional differences between the three patient isolates (Fig 2) (S2 Fig). The separation between patient isolates is especially evident at the early stages of the IDC, where, for instance, the hour 4 post-thaw group has the widest spread, and there is overlap on the PC plot between isolate *Pv*MRMS at hour 4 post-thaw and *Pv*JBC at hour 20 post thaw (Fig 2). However, the general directional trend on the PC plot for each isolate through the IDC over time is consistent, and the transcriptional profiles tend to move toward convergence between isolates at the later developmental stages (Fig 2). Furthermore, the Pearson’s correlation coefficient between isolates at the same time points is markedly high (S3A Fig) Notably, there is negligible separation on the PC plot accounted for by culture media, indicating the media account for relatively little variation in transcriptomic signatures of intraerythrocytic *P*. *vivax* (Fig 2). The minimal influence of culture media on the parasite transcriptomes is also evident when comparing the correlation between media within the same isolate (S3B–S3D Fig).

### Transcriptome of enriched *P*. *vivax* gametocytes

**Fig 3:**
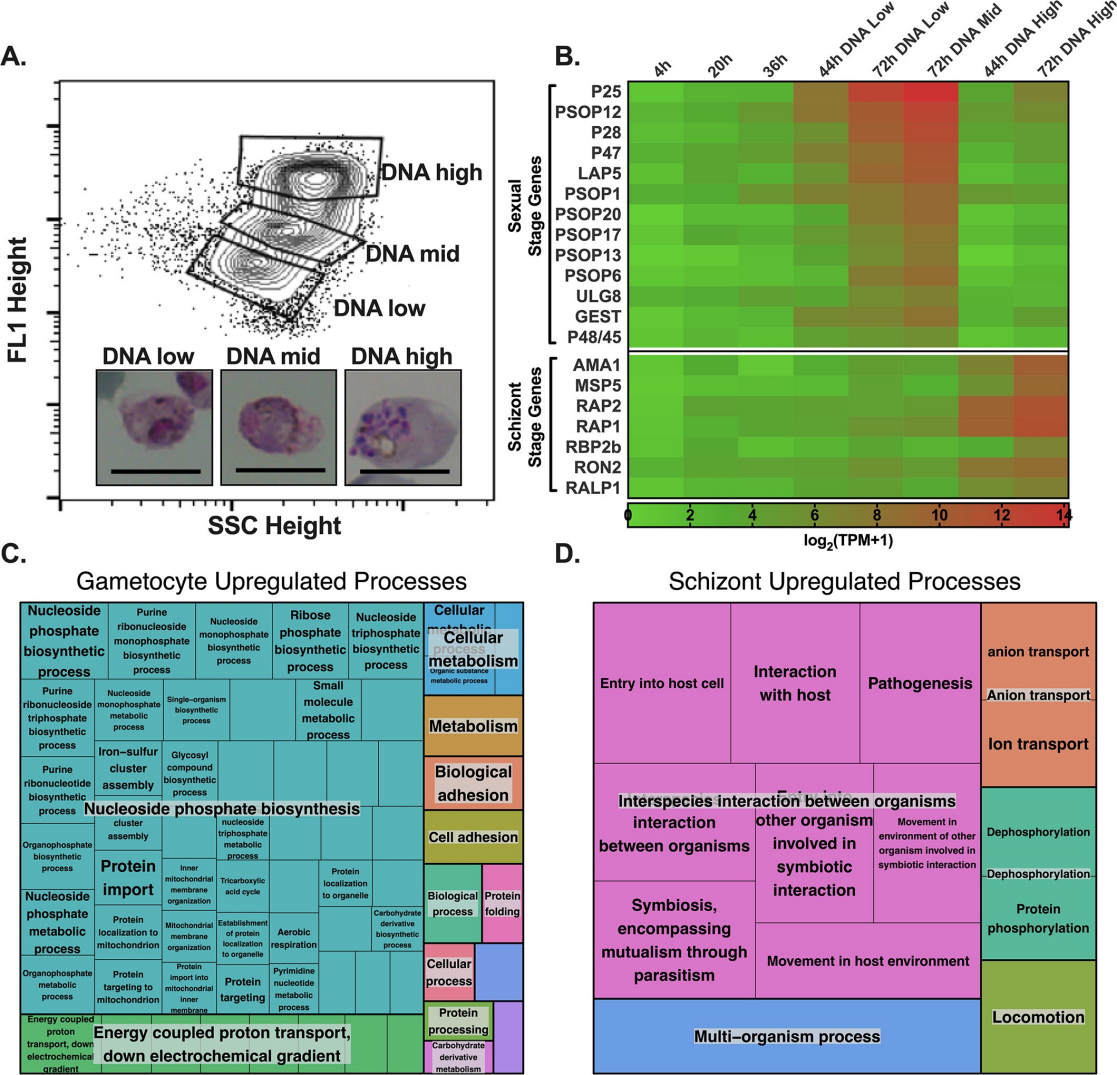
Sorting iRBCs by DNA content at 72 hours post-thaw enables isolation of gametocyte from schizont transcriptomes. A) The gating strategy for DNA-high, DNA-mid and DNA-low FACS-sorted populations after gating for parasite positive cells as in **Fig 1**. Representative images of parasites from each sorted population shown with black bar representing 10 micrometers. B) Heatmap depicting the expression in log_2_(TPM+1) averaged across all available biological replicates of known sexual stage and known schizont stage genes across the intraerythrocytic development cycle. C & D) REVIGO tree maps of gene ontology term biological processes for all genes significantly (Benjamini-Hochberg adjusted p-value < 0.05) upregulated by at least log_2_(fold change) > 2 in the C) gametocyte population or D) the schizont population.

As *ex vivo P*. *vivax* parasites develop from rings to a mixture of schizonts and mature gametocytes *in vitro*, we find that distinct populations of parasites with various levels of DNA content emerge and can be distinguished by flow-cytometry (S4 Fig). For one isolate (*Pv*TCF), enough material was available to extend sampling to 72 hours post-thaw, enabling the fractionation of a sexual gametocyte stage population from the remaining asexual schizont population by FACS (Figs 3A and S4). The relative stage homogeneity of each sort was verified by plotting the averaged expression of known/putative sexual-stage and schizont-stage genes at each time point and sorted population on a heatmap across all biological replicates matured in IMDM [27,28], clearly showing the 72-hour DNA-mid and DNA-low populations represent a primarily sexual transcriptomic profile, while the 72-hour DNA-high sort represents an enriched schizont transcriptomic profile (Fig 3B). Staging of parasites was also performed by microscopy, further confirming enriched gametocyte and schizont populations in the 72-hour DNA-mid and DNA-high populations respectively (S4B Fig). Moreover, there is strong, qualitative agreement between our *P*. *vivax* gametocyte transcriptome and the previously published gamete/zygote transcriptomes, with 12 of their 25 most highly expressed gamete/zygote genes appearing in our top 100 genes most differentially expressed in gametocytes versus schizonts [7].

From these transcriptomes of enriched gametocyte and schizont populations, a list of genes was generated that includes known as well as several previously unattributed genes selectively upregulated in the gametocyte population (Table 1). Some of the most highly expressed genes upregulated in *P*. *vivax* gametocytes, including P25 and Lap5, have already been confirmed as *P*. *vivax* gametocyte markers [29–31]. To provide further, post hoc confidence that the 72-hour DNA-mid sorted cells represent a gametocyte-specific subpopulation, we performed qPCR analysis of expression of the most abundant, differentially expressed gene, P25, identified by our study, as well as, LAP5, finding expression of each is enriched in the 72-hour DNA-mid sample (S5 Fig). Notably, there was failure to amplify all three target genes from the uninfected subpopulation sort, highlighting the successful separation of infected from uninfected host cells.

**Table 1:** Highest expressed genes with ≥ 16-fold increased expression in gametocytes over schizonts.

Gene ID	Gene Name or Symbol	Product Description	Avg TPM 72 hr DNA Mid	Avg TPM 72 hr DNA High	log2(FC)	P value
PVP01_0616100	P25	ookinete surface protein P25	18166	110	6.6	2.93E-08
PVP01_1109400	N/A	Common Plasmodium Protein, Unknown Function	2197	64	4.1	2.96E-08
PVP01_1020200	PSOP12	secreted ookinete protein, putative	5325	61	5.6	5.23E-10
PVP01_1341700	CCp3	LCCL domain-containing protein, putative	2183	58	4.5	6.69E-07
PVP01_0932300	N/A	Common Plasmodium Protein, Unknown Function	1682	55	4.3	2.22E-05
PVP01_0508600	N/A	Common Plasmodium Protein, Unknown Function	3045	36	5.8	6.29E-11
PVP01_1203200	GK	glycerol kinase, putative	1092	31	4.5	1.53E-10
PVP01_1467200	G377	osmiophilic body protein G377, putative	2701	31	5.8	2.46E-11
PVP01_0518500	DMC1	meiotic recombination protein DMC1, putative	2808	27	4.8	1.08E-11
PVP01_0526400	N/A	Common Plasmodium Protein, Unknown Function	1711	24	5.4	1.06E-08
PVP01_1017500	MFS1	major facilitator superfamily domain-containing protein, putative	1837	22	5.6	5.25E-10
PVP01_0616000	P28	ookinete surface protein P28, putative	3437	20	6.5	2.80E-10
PVP01_1208000	P47	6-cysteine protein	1642	20	5.3	8.67E-08
PVP01_0510800	IMC1i	inner membrane complex protein 1i, putative	1163	19	5.1	1.09E-08
PVP01_1345600	N/A	Common Plasmodium Protein, Unknown Function	1903	19	6.0	1.02E-06
PVP01_1433600	N/A	Common Plasmodium Protein, Unknown Function	1512	17	5.6	1.17E-07
PVP01_0508500	N/A	Common Plasmodium Protein, Unknown Function	1193	10	6.0	1.93E-11
PVP01_0517400	HMGB2	high mobility group protein B2, putative	1037	7	5.5	1.50E-15
PVP01_1255400	LAP5	LCCL domain-containing protein, putative	1437	6	7.4	2.09E-09
PVP01_0702600	PH	PH domain-containing protein, putative	1925	2	7.7	8.54E-12

TPM = Transcripts per Million as calucalted by RSEM; FC = Fold Change as calculated by EdgeR; P values are adjusted by the Benjamani & Hochberg method

In addition, tree maps of biological processes corresponding to the gene ontology terms of significantly upregulated genes in the gametocyte and schizont populations were created, showing gametocytes transcripts were mostly devoted to nucleoside phosphate biosynthesis, while schizont transcripts correspond to processes related to interspecies interactions (Fig 3C and 3D).

### Invasion ligand expression

**Fig 4:**
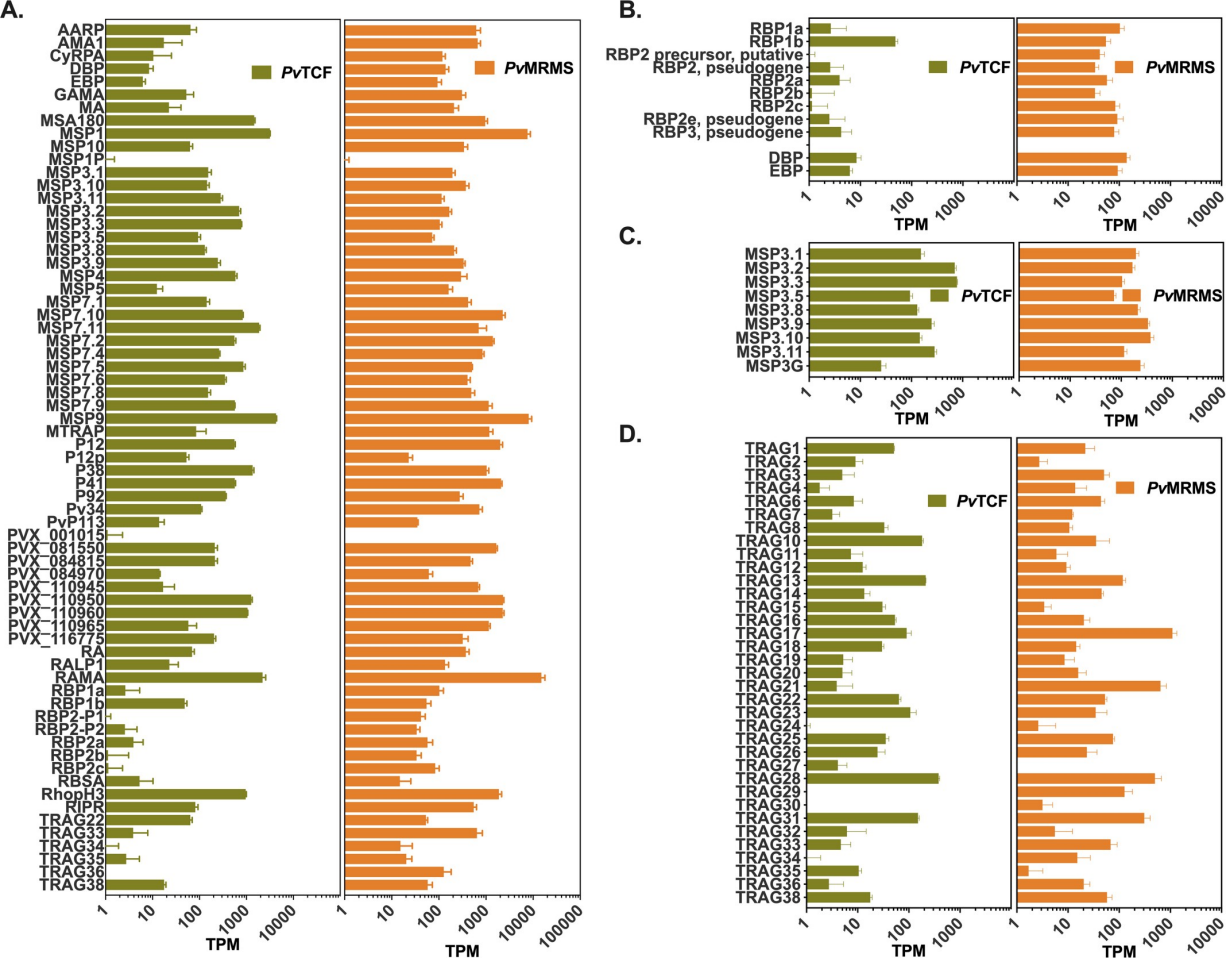
Expression of *P*. *vivax* invasion ligands. Expression levels in transcripts per million (TPM) within isolates *Pv*TCF and *Pv*MRMS at 44 hours maturation in IMDM and FACS sorted by the DNA-high gate of A) confirmed and putatively functional invasion ligands, B) reticulocyte, Duffy, and erythrocyte binding protein (RBP, DBP, and EBP) families, C) merozoite surface protein 3 (MSP3) family and D) tryptophan-rich antigen (TRAG) family. Error bars represent the standard deviation between technical replicates.

Having demonstrated that FACS sorting based on DNA content allowed us to isolate schizont enriched parasite populations, we next sought to investigate the variation in expression of various known or putative *P*. *vivax* erythrocyte invasion ligands and invasion ligand families in the schizont stages, the average TPM of each ligand was plotted for the 44-hour, DNA-high populations of isolates *Pv*TCF and *Pv*MRMS grown in IMDM (Fig 4). This shows a wide variation in invasion ligand expression within an isolate, ranging between 0 and 15000 TPM with ligands MSP1, MSP9, RAMA and RhopH3 in both isolates among the highest expressed putatively functional invasion ligands based on literature evidence [32] (Fig 4A). Also, the general level of invasion ligand expression notably varies between the two isolates, especially among the RBP, MSP3 and TRAG ligand families (Fig 4B–4D). Lastly, to temporally characterize the expression of *P*. *vivax* invasion ligands, the average TPM of each invasion ligand plotted on a heat map for every time point over the intraerythrocytic development cycle for each isolate grown in IMDM, showing that, as expected, the expression of most invasion ligands peaks in the DNA-high populations (S6 Fig).

## Discussion

Though *P*. *vivax* RNAseq transcriptomes through the IDC have been previously generated, these transcriptomes have relied upon clinical samples that can yield relatively large quantities of RNA. The finite nature of clinical samples paired with the characteristic low parasite densities and poor *in vitro* survival of *P*. *vivax*, severely limits the ability to include experimental perturbations in *P*. *vivax* transcriptome studies. This study describes the first use of viable *P*. *vivax* clinical isolates recovered from cryopreservation for the generation of robust transcriptomes using FACS-purified parasites at various stages of maturation throughout the IDC. By combining the Smart-seq2 RNAseq library preparation strategy for low-RNA samples with our recent advancements in *P*. *vivax ex vivo* culture from cryopreserved isolates, we achieve high-quality transcriptomes from just 1000 FACS-purified, infected erythrocytes per sample [5,15]. In addition to establishing a novel *P*. *vivax* RNAseq strategy, we have 1) determined that culture media has relatively minimal effect on transcriptional signatures compared to parasite stage and clinical isolate, 2) generated the first transcriptome of enriched *P*. *vivax* gametocytes and compared it with the transcriptome of enriched *P*. *vivax* schizonts, and 3) examined *P*. *vivax* invasion ligand expression in schizont stages.

RNAseq provides a comprehensive snapshot of the transcriptomic environment of the cells being examined, as opposed to microarray studies, which are inherently targeted. However, because of a lack of *in vitro* culture system for the intraerythrocytic stages of *P*. *vivax*, RNAseq studies for this parasite have been limited by many factors. First, finding or developing the combination of access to fresh clinical isolates and a proximal, well-equipped laboratory to process the samples is particularly challenging and resource-intensive, especially as *P*. *vivax* is most prevalent in resource-poor settings. The use of cryopreserved clinical isolates removes the stipulation of having an experimental laboratory near the clinic; however, to-date no studies have used viable cryopreserved isolates for investigating transcriptomics throughout *P*. *vivax ex vivo* maturation. Indeed, there is only one report of RNAseq analysis over the course of the *P*. *vivax* IDC, and this required the use of relatively large volumes of whole-blood starting material, due to the historical difficulty of *P*. *vivax ex vivo* culture, where parasitemias notably decrease over the course of the IDC [10,11,33]. Though, several groups have recently improved enrichment strategies as well as the survival of *P*. *vivax* through *ex vivo* maturation, we are able to obtain and maintain a higher parasitemia over the IDC, allowing the use of much less starting material for experimental studies [5,34].

An additional complication that often accompanies transcriptomic studies of *P*. *vivax* from whole blood samples is the prevalence of human transcripts in the RNA library, which can reduce the sensitivity of sequencing to parasite reads. 89–96% of our generated reads map directly to the *Pv*P01 genome, indicating a very low quantity of contaminating human RNA in our preparations. This is likely because our process includes an established, robust but simple leukodepletion method prior to cryopreservation, and because we FACS-purified infected erythrocytes by DNA content before RNA isolation. With total read numbers between 5 and 10 million mapped reads per sample, we get similar results to previous *P*. *vivax* RNAseq experiments using small-volume isolates [6], and our transcriptome saturation curves all plateau, indicating the dataset has captured the vast majority of genes expressed for each sample. Important to note is that excessive preamplification of the cDNA could create systematic bias in the reads detected, and that while other *Plasmodium* transcriptomic studies have utilized up to 30 rounds of PCR preamplification, this study only used 18 cycles, reducing the chance for bias [35,36]. Another important contrast is that most previous *P*. *vivax* RNAseq studies have chosen to use the Salvador-I genome as a reference, but our use of the newer *Pv*P01 reference genome may assist in generating more robust data and mapping efficiency, because the *Pv*P01 genome is a nearly 10% larger assembly with much deeper fold-coverage and 22% more genes than Salvador-I, largely in the subtelomeric regions [18].

Although it is clear that culturing *P*. *vivax* in IMDM enables the parasites to survive much better than when cultured in formulaically similar Dulbecco’s Modified Eagle’s Medium (DMEM) or in the current *P*. *vivax* standard, McCoy’s 5A, the causal formulation components remain unclear [5]. We opted to test the effect of these various media as well as another hematopoietic cell culture medium, Aim V, on the transcriptomic profiles of intraerythrocytic *P*. *vivax*, with the goal of identifying gene expression responses that may elucidate the biological processes better supported by IMDM. The PC analysis does clearly show that the vast majority of variation in transcriptomes is due to parasite stage, which is expected as *Plasmodium spp*. transcriptional signatures are known to vary widely across the IDC [37]. Additionally, the variation accounted for by clinical isolate at early stages of the IDC, is also anticipated for two reasons. The first explanation for the spread in data in the early-stage parasites is that it has been previously noted across many *Plasmodium* species that there is relatively large transcriptional variation between early stage intraerythrocytic parasites, which is hypothesized as a mechanism by which newly-invaded parasites adapt to heterogeneous host cell environments [37]. A second reason we expect wide transcriptional variation in the early-stage parasites is, although only the *P*. *vivax* parasites in the early stages of the IDC tend to survive our cryopreservation process [5], the isolates from separate patients are still likely to represent a range of early stage parasites. Because these are primary patient isolates, it is impossible to control for the range of early stages present at the time of sample collection. Indeed, we find that there is marked overlap on this PC plot between the 4-hour *Pv*MRMS samples with the 20-hour *Pv*JBC samples, which we interpret as the parasites from the *Pv*JBC isolate being much younger than the parasites from *Pv*MRMS at thaw (Fig 2). Nonetheless, each isolate ultimately follows similar transcriptomic paths through the IDC, which is supported by the observation that the general trajectory of each biological replicate is similar in the two-dimensional PC analysis space as the IDC progresses (Fig 2). Remarkably, there is little separation in the PC analysis accounted for by culture media, and we found no significant, biologically reproducible, transcriptomic signature distinguishing parasites grown in IMDM versus to other media. Although they do not help define the mechanism of differential growth in various media formulations, these results may indicate a lack of transcriptional flexibility of *P*. *vivax* at each time point of the IDC, which could help explain the relatively fastidious nature of *P*. *vivax ex vivo* culture. Moreover, from our previous study, it is clear that when the parasites do die in each media, they are not visible as pyknotic cells, but rather disappear entirely, suggesting a red blood cell-focused hemolytic event [5]. Thus, the lack of transcriptional response to various media may indicate the media are directly affecting the host cell itself, resulting in either hemolysis or relative stability; however, additional studies are required to parse the role of the host cell in *P*. *vivax in vitro* maturation.

Previously, the transcriptomes of enriched *P*. *vivax* gametes, zygotes and ookinetes have been characterized through microarray analysis [7], but an RNAseq-based transcriptome of the enriched intraerythrocytic *P*. *vivax* gametocytes has not been achieved, again, largely due to the difficulty in generating sufficient gametocyte material for RNA library generation. Because we utilize viable *P*. *vivax* parasites recovered from cryopreservation, we are able to perform our experiments geographically separated from the largely resource-poor, endemic area from which the samples are obtained. This enabled us to capitalize on the state-of-the-art facilities and resources available to us, maximizing the viability of the parasites, even out to 72 hours post-thaw for one isolate, *Pv*TCF, enabling the separation of enough gametocytes from schizonts to achieve robust transcriptomic information from three technical replicate sorts for each parasite subpopulation. It remains unclear why there are persistent schizont parasites at this late time point. Possible explanations could be a large heterogeneity in the maturation time of asexual *P*. *vivax* parasites, or the potential stall of maturation and subsequent reactivation, a phenomenon which has been considered previously [33]. However, the 72-hour DNA-high parasites cluster very closely to the 44-hour DNA-high parasites on the PC plot, suggesting that these two populations are transcriptionally quite similar despite their temporal separation, providing confidence that this population represents schizonts, enabling the generation of a compressive list of genes that are selectively upregulated in gametocytes compared to schizonts. Qualitatively, this list of putative *P*. *vivax* gametocyte markers has many proteins previously implicated in *Plasmodium spp*. gametocyte biology including G377, Lap5, and P25 [7,29–31,38]. Interestingly, this list also includes PSOP12, which has been shown to induce transmission blocking immunity in *P*. *berghei* mouse models [39,40]. Other, uncharacterized genes in this list may also be suitable antimalarial or vaccine targets in the future.

In terms of invasion ligand expression, though there is notable variation within an isolate, we find highly antigenic ligands, such as MSP1, MSP9, MSA180 and RAMA, are most highly expressed, which is encouraging because each of these is also being considered for vaccine development [41–43]. It is important to note, however, that while there is marked differences in invasion ligand expression between the isolates, an unknown portion of this variation is likely explained by the differences in the maturity of the schizonts, making it difficult to compare across biological replicates. Future studies should be performed with sufficient temporal sampling resolution to enable the employment of established statistical methods to accurately estimate the maturation state of the parasites [44]. Additionally, future RNAseq studies using many more isolates from geographically distinct regions would be beneficial in characterizing the full diversity of invasion ligand expression by the *P*. *vivax* parasite, especially among multigene families like the MSP3s, TRAGs or RBPs. Lastly, employing the growing ability of *Plasmodium spp*. single-cell transcriptomic approaches [35,45] would be quite helpful in characterizing the variation of invasion ligand expression between parasites within an isolate, which would help determine whether such variation is more apparent between infections or between parasites within an infection.

In summary, this study provides strong evidence that small volume, cryopreserved *P*. *vivax* isolates can be used to attain robust RNAseq information, enabling the acquisition and analysis of transcriptomic information at research sites geographically removed from clinical sites of sample acquisition. Furthermore, this approach reduces experimental variability across biological replicates as transcriptomic studies from various cryopreserved *P*. *vivax* isolates can be performed in batch rather than sequentially as live isolates present in the clinic. This study reports the FACS-based isolation of gametocytes from asexual parasites, allowing us to generate the first transcriptome from enriched *P*. *vivax* gametocytes and to compare the expression of various invasion ligands in enriched *P*. *vivax* schizonts. Utilizing these FACS-based stage isolation methods, we recommend future studies generate schizont transcriptomes from many, isolates to more robustly investigate the differential expression of invasion ligands between infection to better inform *P*. *vivax* vaccine development. Moreover, although we confirm large differences in transcriptome profiles between patient isolates, this is likely due to differences in the parasite staging at time of sample acquisition, which could be resolved in the future utilizing statistical methods on data with higher temporal resolution [44]. Such experiments could help to inform the development of novel vaccines, chemotherapeutics and experimental techniques. Indeed, this present study empowers such *P*. *vivax* transcriptomic experiments.

## Supporting information

S1 Fig A threshold of TPM = 1 for gene expression is empirically reasonable. Density curve of the calculated gene expression represented as log_2_(TPM+0.1) across all technical and biological replicates with the vertical dotted red line representing the TPM = 1 threshold for categorizing a gene as expressed.
(TIF)

S2 Fig The majority of transcriptional variation is explained the first two principle components. A scree plot showing the proportion of variance in gene expression explained by each of the top ten principle components generated.
(TIF)

S3 Fig There is strong positive correlation of transcriptomes across biological replicates and across culture media. A) Heatmap representing the calculated Pearson’s Correlation Coefficient (PCC) between *P*. *vivax* transcriptomes of biological replicates and time points cultured in IMDM. B-D) Heatmaps of the calculated PCC between *P*. *vivax* transcriptomes of parasites in various culture media and time points.
(TIF)

S4 Fig Flow cytometry and representative microcopy image of *P*. *vivax* parasites throughout the intraerythrocytic development cycle. A) Flow cytometric plots of gate-purified *P*. *vivax* (Fig 1B) comparing DNA content by Vybrant DyeCycle Green stain and side scatter at each sampled time point for isolate *Pv*TCF grown in IMDM. B) *P*. *vivax* staging and representative images of PvTCF parasites from each time point. Black bars within images represent 10 micrometers.
(TIF)

S5 Fig The 72-hour DNA-mid sort is enriched for sexual-stage-specific transcripts. Gene expression levels of P25 (PVP01_0616100) and LAP5 (PVP01_1255400) normalized initially to MRScyt (PVP01_0620000) then to the 72-hours DNA-high samples.
(TIF)

S6 Fig Expression of putatively functional *P*. *vivax* invasion ligands varies across intraerythrocytic development. Heatmap depicting the average invasion ligand expression in log_2_(TPM+1) by isolates grown in IMDM across the intraerythrocytic development cycle.
(TIF)

S1 Table RT-qPCR primers. Primer nucleotide sequences used for RT-qPCR validation gametocyte enrichment within the 72-hour DNA-mid sorted cell population.
(XLSX)
