# Supplementary figures and images for: Plasmodium vivax transcriptional profiling of low input cryopreserved isolates through the intraerythrocytic development cycle
Source: PLoS Negl Trop Dis. 2020 Mar 2;14(3):e0008104. doi: 10.1371/journal.pntd.0008104 (PMC7067476; doi:10.1371/journal.pntd.0008104)

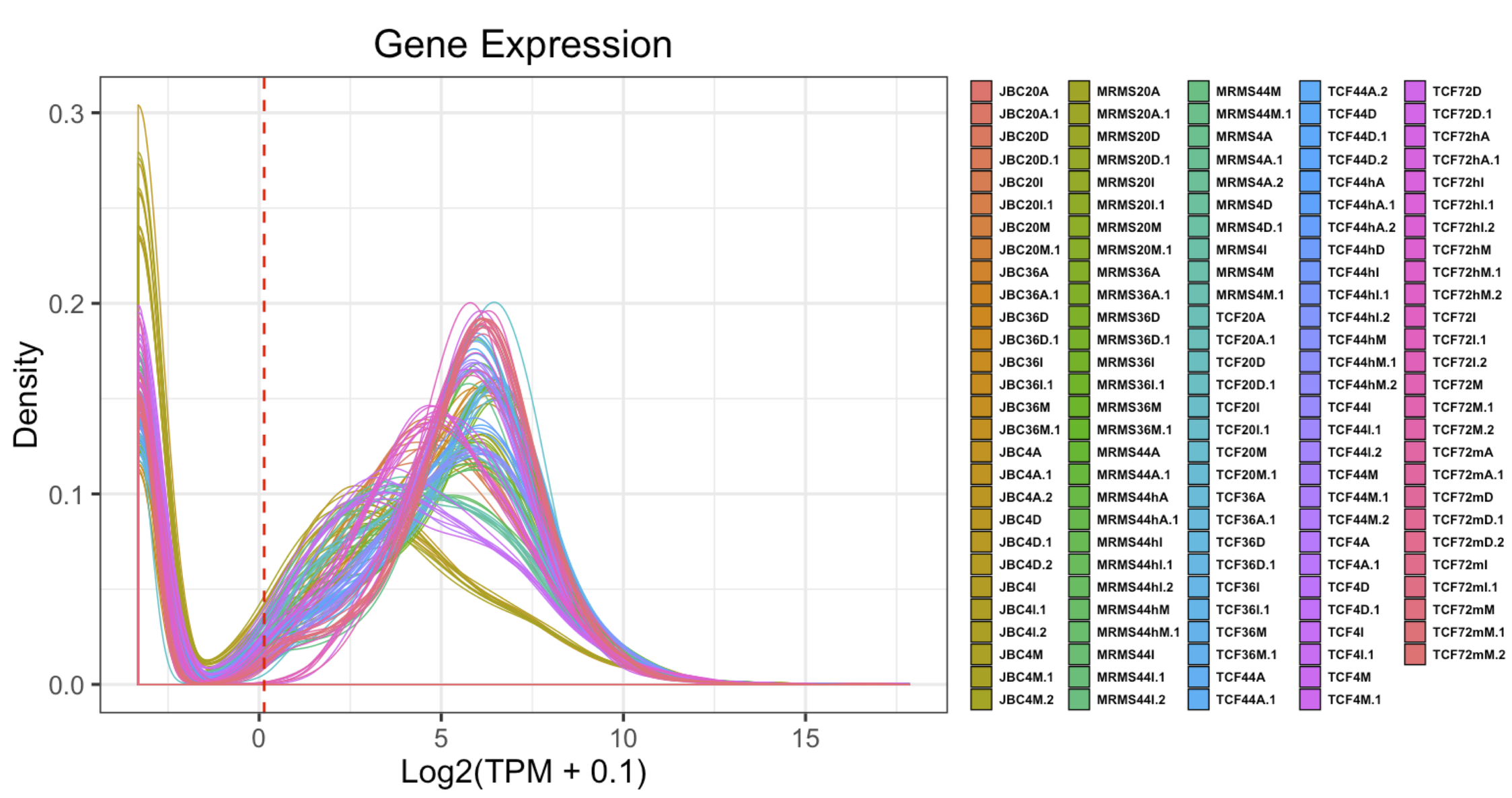

Supplement: S1 Fig — Density curve of the calculated gene expression represented as log2(TPM+0.1) across all technical and biological replicates with the vertical dotted red line representing the TPM = 1 threshold for categorizing a gene as expressed. (TIF) [file pntd.0008104.s001.tif]

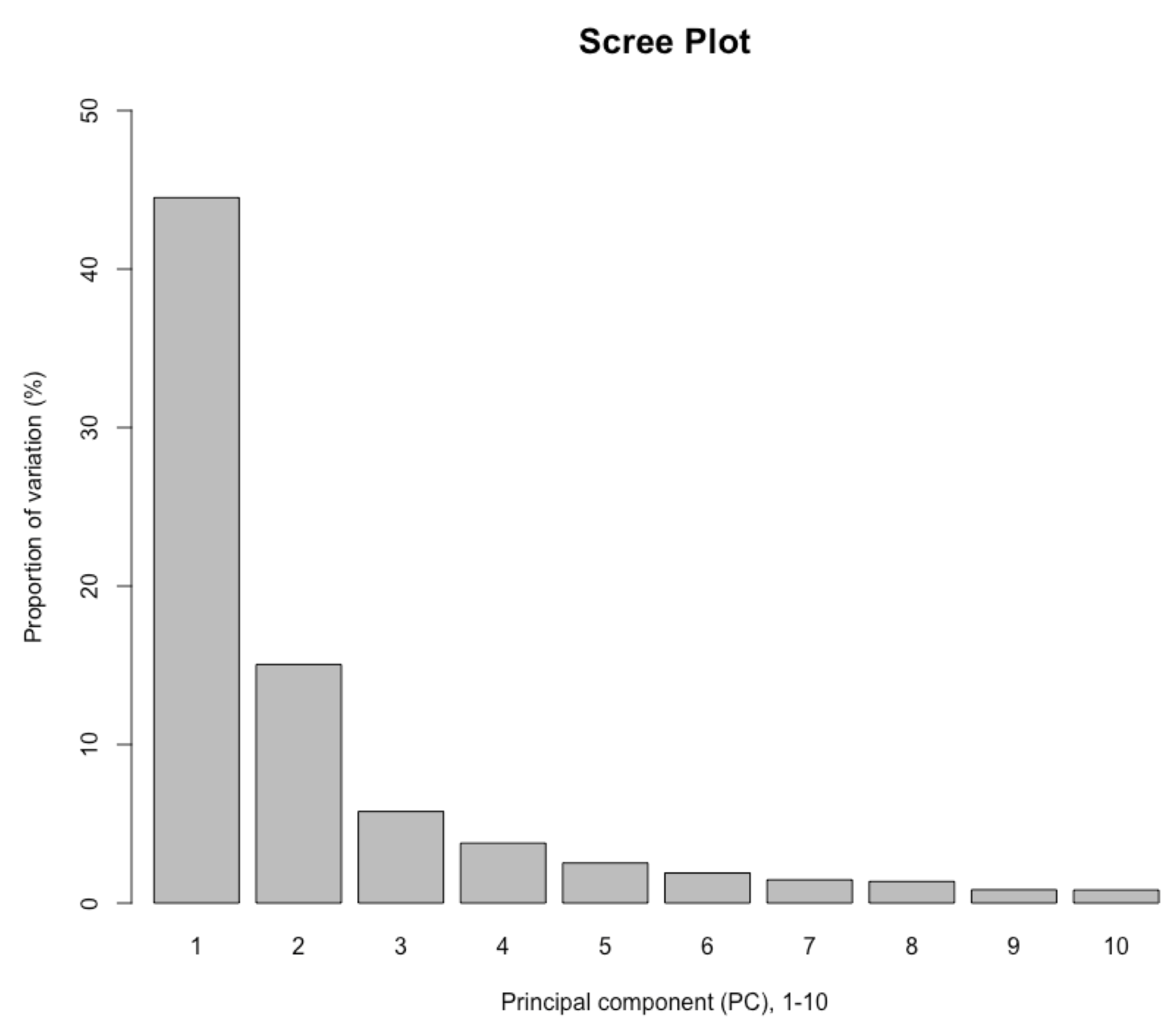

Supplement: S2 Fig — A scree plot showing the proportion of variance in gene expression explained by each of the top ten principle components generated. (TIF) [file pntd.0008104.s002.tif]

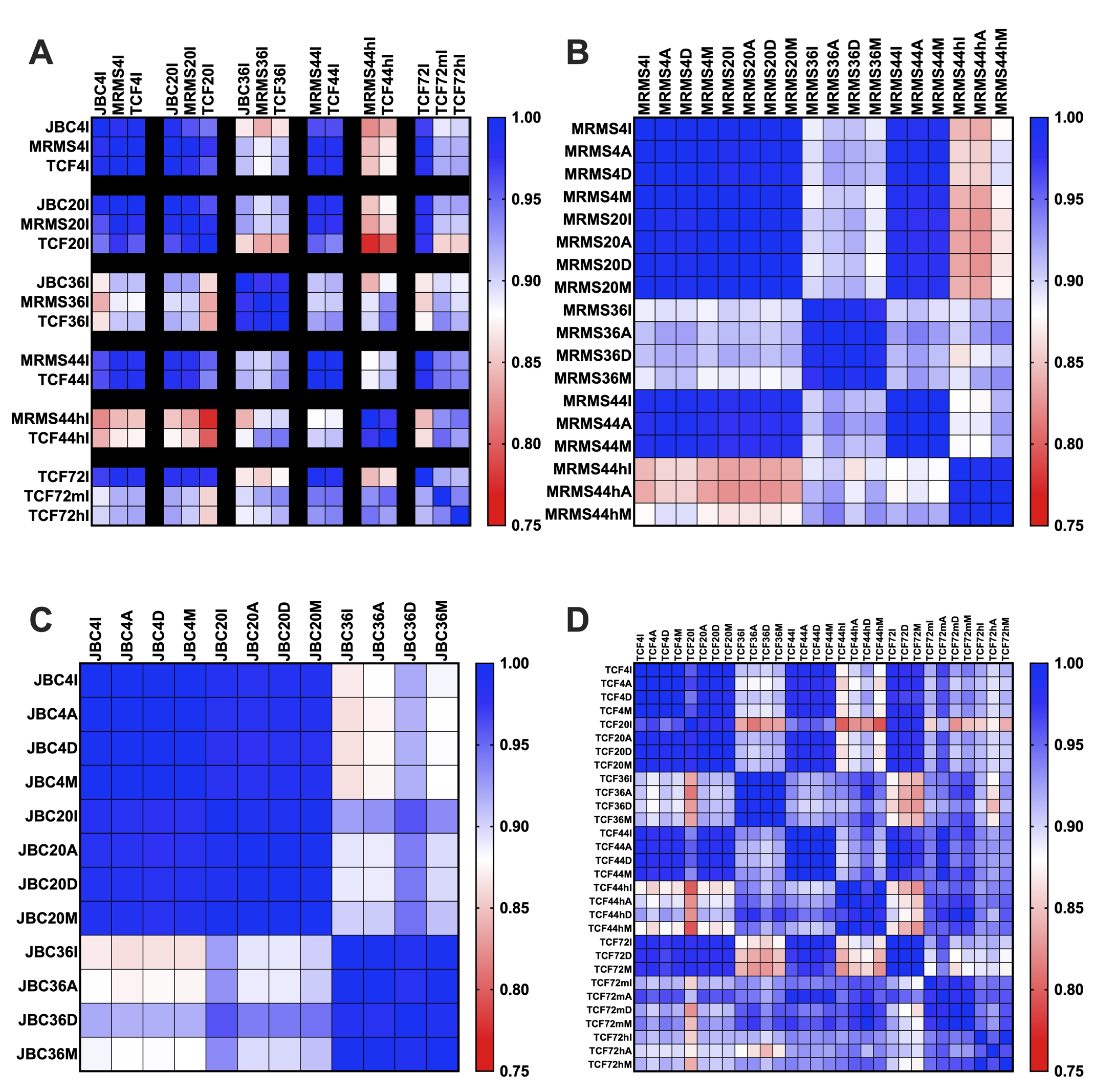

Supplement: S3 Fig — A) Heatmap representing the calculated Pearson’s Correlation Coefficient (PCC) between P. vivax transcriptomes of biological replicates and time points cultured in IMDM. B-D) Heatmaps of the calculated PCC between P. vivax transcriptomes of parasites in various culture media and time points. (TIF) [file pntd.0008104.s003.tif]

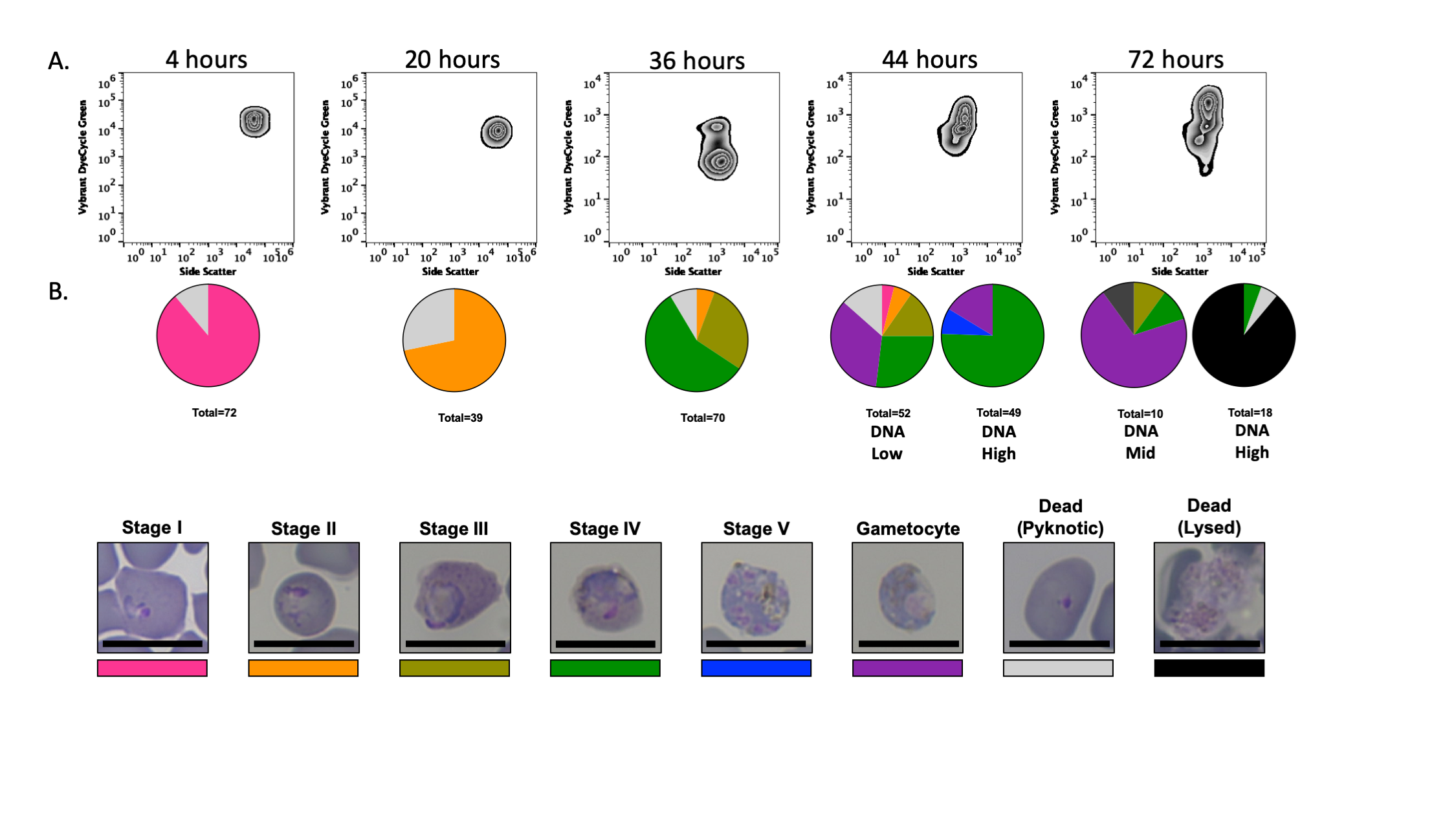

Supplement: S4 Fig — A) Flow cytometric plots of gate-purified P. vivax (Fig 1B) comparing DNA content by Vybrant DyeCycle Green stain and side scatter at each sampled time point for isolate PvTCF grown in IMDM. B) P. vivax staging and representative images of PvTCF parasites from each time point. Black bars within images represent 10 micrometers. (TIF) [file pntd.0008104.s004.tif]

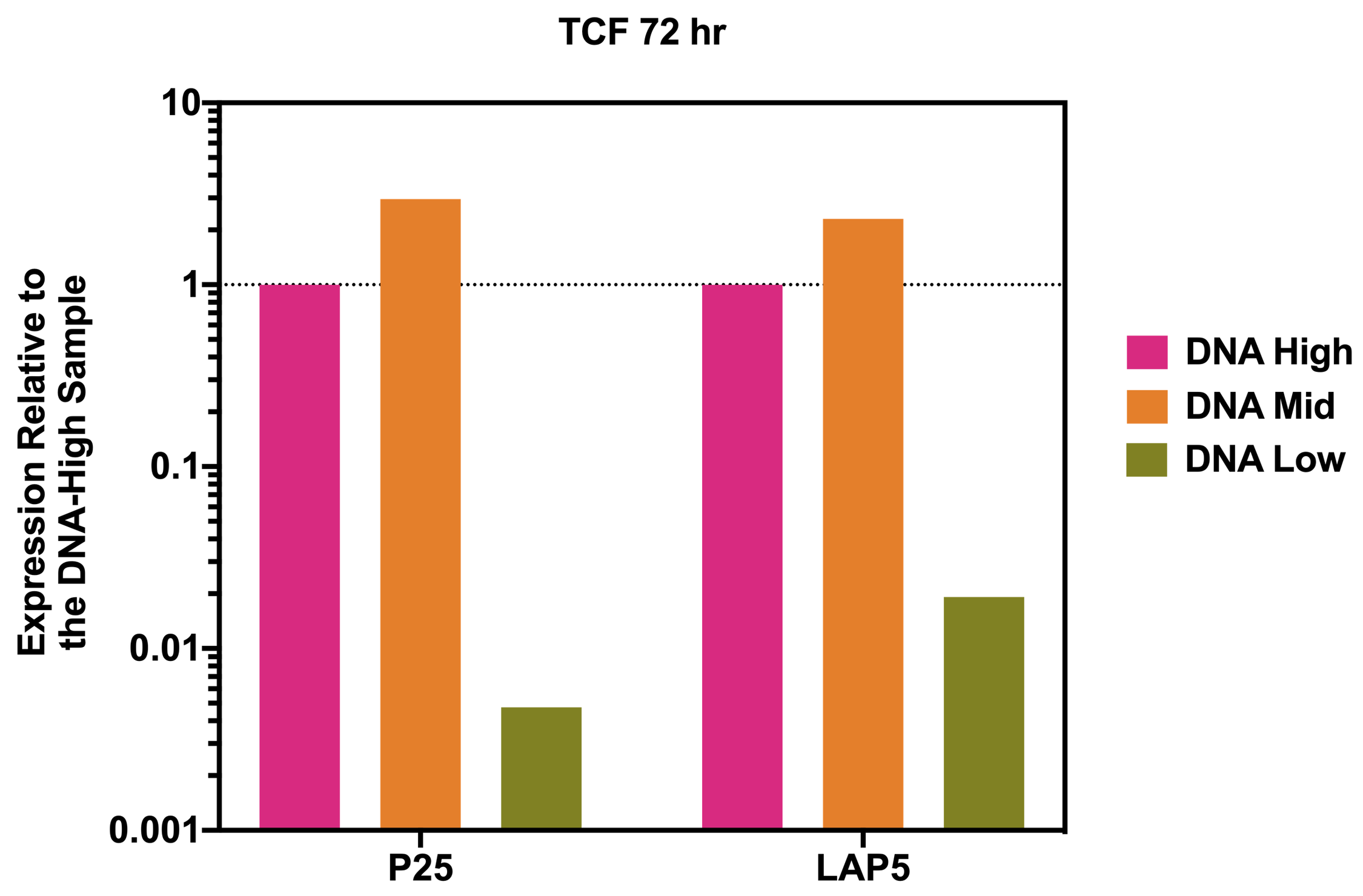

Supplement: S5 Fig — Gene expression levels of P25 (PVP01_0616100) and LAP5 (PVP01_1255400) normalized initially to MRScyt (PVP01_0620000) then to the 72-hours DNA-high samples. (TIF) [file pntd.0008104.s005.tif]

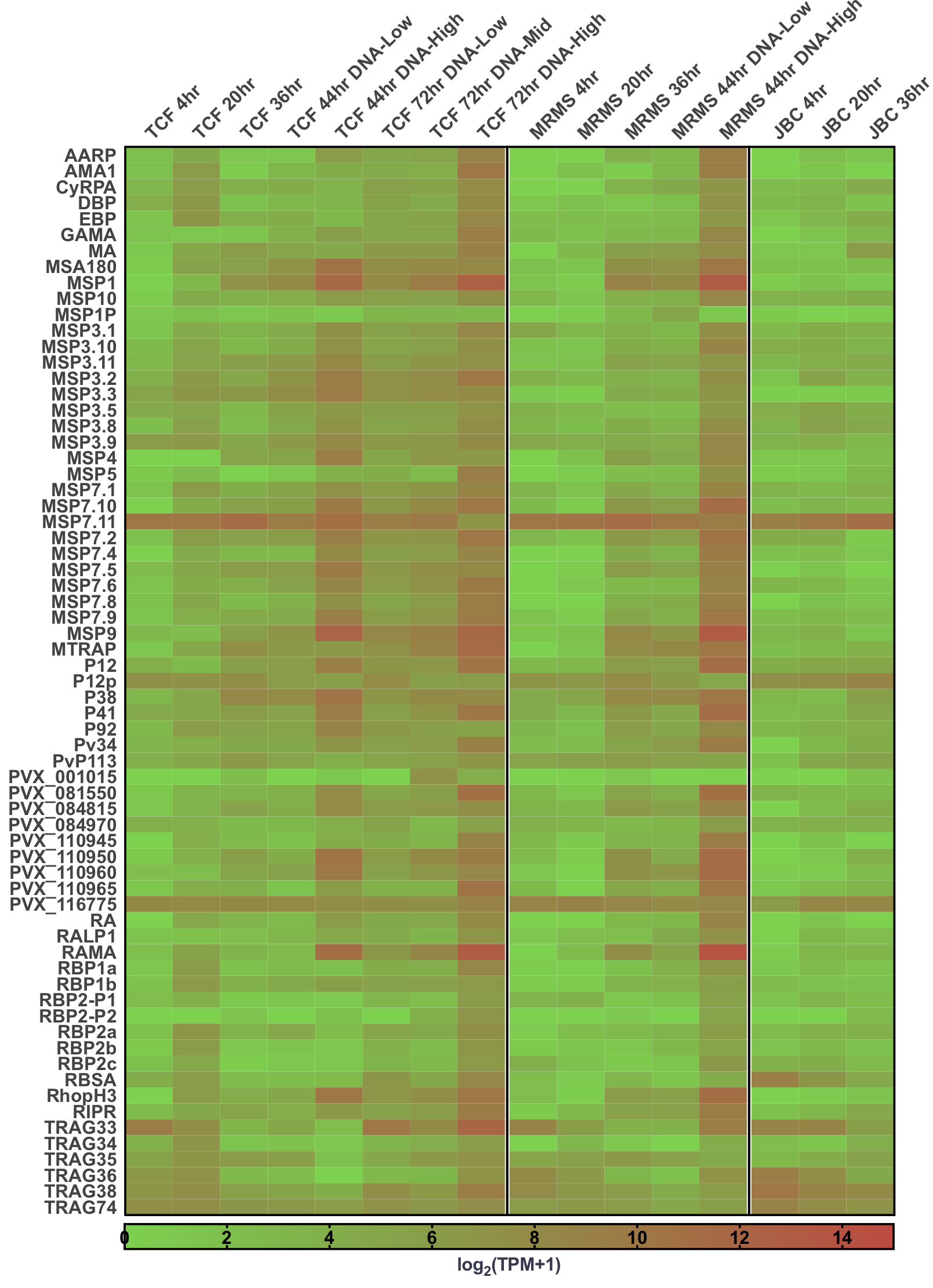

Supplement: S6 Fig — Heatmap depicting the average invasion ligand expression in log2(TPM+1) by isolates grown in IMDM across the intraerythrocytic development cycle. (TIF) [file pntd.0008104.s006.tif]
